# Importance of occupational dentistry for companies - a literature review

**DOI:** 10.47626/1679-4435-2021-644

**Published:** 2021-12-30

**Authors:** Izabel Cristina Leite Albuquerque, Joanne Bezerra Rodrigues, Tayane Soares de Melo Vieira, Rafaela Cavalcanti Amaral, Mariana Passos

**Affiliations:** 1 Centro Universitário UNINASSAU, Aracajú, SE, Brazil.; 2 Centro Universitário Tiradentes, Maceió, AL, Brazil.; 3 Faculdade São Leopoldo Mandic, Campinas, SP, Brazil.; 4 Universidade Estácio de Sá, Rio de Janeiro, RJ, Brazil.

**Keywords:** occupational dentistry, oral health, company, odontologia do trabalho, saúde bucal, empresa

## Abstract

Due attention has not been given yet to workers’ health problems arising from oral diseases and to the impacts of not having an appropriate professional, i.e., dental surgeon, as part of the specialized service team of large companies, even acknowledging that the participation of these professionals is positive for improving workers’ quality of life and the productive performance of the company. Based on these premises, and through a literature review conducted from a search on the electronic portal of Brazilian Dentistry Bibliography, SciELO, and MEDLINE databases, the present study aimed to demonstrate factors that justify the integration of dental surgeons in occupational health programs of companies. The following advantages were mentioned: reduction in absenteeism and in the number of work accidents, productivity growth, and improvement in company image and in employees’ quality of life. In conclusion, the occupational health care model cannot be dissociated from oral health, considering the significant benefits both for employees and for companies themselves.

## Resumo

Ainda não foi dada a devida atenção aos problemas de saúde do trabalhador decorrentes das doenças bucais nem aos reflexos de quando o profissional adequado, o cirurgião-dentista, ainda não faz parte da equipe do serviço especializado das grandes empresas, mesmo se reconhecendo sua participação positiva para a melhoria da qualidade de vida do trabalhador e para o melhor desempenho produtivo da empresa. Com base nessas premissas e por meio de uma revisão de literatura realizada a partir da busca literária no portal eletrônico das bases de dados Bibliografia Brasileira de Odontologia, SciELO e MEDLINE, o presente trabalho teve como objetivo demonstrar fatores que justificassem a inserção do cirurgião-dentista no programa de saúde do trabalhador na empresa, citando como vantagens a diminuição do absenteísmo e do número de acidentes de trabalho, o aumento da produtividade e a melhoria da imagem da empresa e da qualidade de vida dos funcionários. Conclui-se que o modelo de atenção de saúde do trabalhador não pode estar dissociado da saúde bucal, tendo em vista o significativo número de benefícios tanto para o empregado como para a própria empresa.

## INTRODUCTION

Since work is a necessary benefit for maintenance of life based on creation and productivity, it becomes extremely important to maintain worker’s health status for the successful execution performance.^1,2^ Occupational health has shifted from practices grounded in the medical context to practices based on health promotion and preservation with the organization of interdisciplinary teams, into which the work of occupational dental surgeons is integrated, with the aim of improving workers’ physical and emotional conditions.^1,3^ However, there is a gap in occupational oral health care.

Exposure to occupational risks when in contact with physical, chemical, and bacteriological agents has become a determining factor to justify the presence of occupational dentists in occupational health programs.^4^

Dentistry should occupy its rightful position in occupational health, given the diversity of occupational diseases that lead to oral manifestations. This area of knowledge is responsible for the performance of dental examinations for labor purposes and for the provision of technical assistance and care in matters relating to occupational health, safety, ergonomics, and hygiene, as well in those relating to personal protection equipment, and occupational dentists are integrated into an interdisciplinary occupational health team, complying with the areas of competence defined by the 3^rd^ article of resolution 25/2002 of the Federal Council of Dentistry (Conselho Federal de Odontologia, CFO).^5^

Therefore, the present study aims to point out the relevance of the role of dental surgeons in the occupational health program of companies, in view of the need to value human resources and to maintain the quality of life of the working population.

## PROPOSITION

This study aimed to perform a non-systematic literature review on the occupational dentistry specialty, in order to emphasize the need for prevention and for measures of workers’ oral health care in companies.

## METHODS

This study was conducted using the bibliographic research method, which consisted of search on the web portals of Brazilian Dentistry Bibliography, SciELO, and MEDLINE databases, employing the following descriptors: occupational dentistry; oral health; company. The selected articles met the inclusion criteria, which aimed to select current journals, articles specifically on occupational dentistry, and studies that addressed oral changes in workers, assessments of decrees and updates of bills, thus excluding the remaining articles.

### LITERATURE REVIEW

Studies have already proven that tooth decay and other oral complications are responsible for 20% of work absences and decline in production, in addition to causing other types of organic complications, such as sepsis. This is called absenteeism due to physical absence by the World Health Organization and is exemplified when employees are unable to concentrate in their activities because they have a toothache.^6,7^

Occupational risks are known to be dependent on the environmental problem; thus, the presence of a health care professional in companies is beneficial. Since workers are in frequent contact with chemical, physical, biological, and psychological agents that may cause accidents or diseases, they rely on health care professionals as attentive observers who identify potential hazards and implement control measures.^8^

Occupational dentistry is focused on the study of health risks for the oral and maxillofacial complex resulting from work practice, as well as on the implications of dental diseases and conditions on labor issues.^5^ Of many benefits, such as preventive and early approach to provide or facilitate workers’ access to oral health, the most predominantly observed is improvement in physical, mental, and social wellbeing, with consequent reduced absenteeism and productivity growth.^6,9^

With the investment in oral health, the company will become more valuable in the market, resulting in increased individual productivity and decreased likelihood of work accidents and occupational diseases with oral manifestations. Reduced rates of absenteeism, work accidents, and occupational diseases collaborate for an improvement in national production and in the supply of industrialized products.^3,10,11^

The company itself also benefits from the inclusion of occupational dental surgeons in occupational health outpatient clinics, due to decreased rates of absenteeism.^11^

In September 2001, the CFO approved the new occupational dentistry specialty, aiming for the permanent pursuit of compatibility between labor activity and preservation of worker’s oral health.^12^ Resolution CFO 116/2012 states the following:

“Their competencies are: a) To identify, assess, and monitor the environmental factors that may represent a risk for oral health in the workplace, in any phase of the production process; b) To provide technical assistance and care in matters relating to occupational health, safety, ergonomics, and hygiene, as well in those relating to personal protection equipment, being considered integrated into the interdisciplinary occupational health team; c) To plan and implant permanent campaigns and programs to educate workers about occupational accidents, occupational diseases of the oral cavity, and health education; d) To organize statistics on morbidity and mortality from oral causes and to investigate their possible association with labor activities; e) To perform dental examinations for labor purposes; and f) To conduct a social and epidemiological analysis of workers’ oral health problems.”^12,13^

The Bill 422/2007, which address the integration of the dental surgeon specialized in occupational dentistry into the occupational health team, is still under consideration by commissions in the House of Representatives.^14^

Absenteeism leads to increased financial costs, reduced productivity and efficiency, and increased administrative problems, compromising the industrial system. With economic and technological advances in society, it becomes necessary to work more effectively to reach the maximum industrial efficiency. Workers who are professionally satisfied have greater qualification and agility, contributing to the actual development of the company.^15,16^

A study on absenteeism due to dental reasons according to position or occupation of municipal civil servants in Guarulhos, Brazil, revealed that an average of 1.55 workdays were lost by employees per year, after assessing 1,015 registered medical certificates in a population of 15,625 servants in 2007. This research showed the relevance of including oral health in occupational health programs to reduce absenteeism and to improve workers’ quality of life, consequently promoting a positive effect for companies.^16^

The possible contributions of occupational dentistry in workers’ performance and quality of life are reduced absenteeism and decreased work accidents, which inevitably leads to a productivity gain. Poor oral health conditions may cause absenteeism or productivity decline in a company, because workers with toothache not only lose concentration, for example, facilitating the occurrence of accidents and technical errors, but also experience mood and behavioral changes, impairing interpersonal relationships. Therefore, the benefits arising from the integration of dental surgeons into occupational health teams include from leaning oral hygiene care measures, increasing workers’ drive, and improving worker’s image of the company to facilitating access to dental care, treating oral diseases, and eliminating infectious foci.^17,18^

The epidemiological principles are essential to produce knowledge on the occupational health field, into which occupational dentistry is inserted. Successful experiences of implantation of workers’ oral health care programs in companies of the industrial sector industrial showed the need for a greater integration of dental professionals into health care and work safety team, in order to control risk factors present in the workplace that are associated with changes in oral tissues.^18,19^

The most effective and determining form of action of occupational dentistry is preventing changes in oral tissues and reducing the need of urgent treatment, with a consequent reduction in absenteeism, since studies indicate that toothache is the leading type of pain causing work absences.^20^

The participation of dental surgeons in oral health programs targeted at workers should especially aim to promote, protect, recover, and rehabilitate the oral health of these workers, thus contributing for the improvement of their quality of life.^21,22^

## DISCUSSION

Absenteeism is one of the main problems found during work at companies, since the participation of all workers is crucial during work execution in order for it to be developed efficiently. The literature described two forms of absenteeism during production processes, which are named as physical absenteeism, when employee’s absence is required, and presenteeism, when employees are in their workplace but do not perform their role in an efficient manner, resulting in a production decline.^6,11,17,21^

The role of occupational surgeon dentists, similar to that of occupational physicians, is both treating the problem presented by workers and preventing, informing, and assessing the working conditions of these patients, so as to prevent future problems, in addition to solving already existing problems, since workers are vulnerable to possible harms from contact with physical, chemical, and biological agents, depending on the work environment.^8,19^

Law 5,081 regulates the practice of dental surgeons, who may work in the oral health field together with an interdisciplinary team in the occupational health context.^9,14,17,19,23^

The participation of dentistry in the work of companies will promote benefits not only for worker but also for companies, since it fosters production, because early diagnosis of oral changes will reduce absenteeism. Based on the assessment of workers’ oral health, other harms resulting from exposure to chemical, physical, and biological agents should be assessed by dental surgeons. They should diagnose and preventively intervene to minimize risks during work activities.^4,16,17^

Since occupational dentistry aims to contribute for the better development of actions of oral health prevention and promotion, which are an integral part of occupational health, Bill 422/2007 was formulated, aiming to mandatorily include the participation of dental surgeons in occupational health teams.^9,14,24^

## CONCLUSIONS

The integration of dental surgeons as team members of the occupational health control program of companies is essential for promoting, protecting, and recovering workers’ oral health, thus reducing the need for more invasive treatments and, consequently, absenteeism. The inclusion of these professionals aims to improve employees’ quality of life, thus resulting in work improvement and, consequently, in the valuing and development of companies. Therefore, the benefits of dental surgeons’ participation in occupational health are unquestionable. However, there are still no plans for the legal integration of these professionals into the team of Specialized Services in Safety Engineering and Occupational Medicine (Serviços Especializados em Engenharia de Segurança e em Medicina do Trabalho), since Bill 422/2007 is waiting for approval in the House of Representatives.
